# Integrated Untargeted Metabolome, Full-Length Sequencing and Transcriptome Analyses Reveal the Mechanism of Flavonoid Biosynthesis in Blueberry (*Vaccinium* spp.) Fruit

**DOI:** 10.3390/ijms25084137

**Published:** 2024-04-09

**Authors:** Youwen Tian, Xinlei Liu, Xuyang Chen, Bowei Wang, Mei Dong, Li Chen, Zhengsong Yang, Yadong Li, Haiyue Sun

**Affiliations:** 1 College of Horticulture, Jilin Agricultural University, Changchun 130118, China; tianyouwen5219@163.com (Y.T.); liuxinlei0202@163.com (X.L.); 15004917050@163.com (X.C.); wbw0150@163.com (B.W.); chenli@jlau.edu.cn (L.C.); 2 College of Life Sciences, Jilin Agricultural University, Changchun 130118, China; dongmei4790@163.com; 3 High Mountain Economic Plant Research Institute, Yunnan Academy of Agricultural Sciences, Lijiang 674110, China; muzi91_1@163.com

**Keywords:** blueberry, metabolome, SMRT sequencing, transcriptome, flavonoids

## Abstract

As a highly economic berry fruit crop, blueberry is enjoyed by most people and has various potential health benefits, many of which are attributed to the relatively high concentrations of flavonoids. To obtain more accurate and comprehensive transcripts, the full-length transcriptome of half-highbush blueberry (*Vaccinium corymbosum/angustifolium* cultivar Northland) obtained using single molecule real-time and next-generation sequencing technologies was reported for the first time. Overall, 147,569 consensus transcripts (average length, 2738 bp; N50, 3176 bp) were obtained. After quality control steps, 63,425 high-quality isoforms were obtained and 5030 novel genes, 3002 long non-coding RNAs, 3946 transcription factor genes (TFs), 30,540 alternative splicing events, and 2285 fusion gene pairs were identified. To better explore the molecular mechanism of flavonoid biosynthesis in mature blueberry fruit, an integrative analysis of the metabolome and transcriptome was performed on the exocarp, sarcocarp, and seed. A relatively complete biosynthesis pathway map of phenylpropanoids, flavonoids, and proanthocyanins in blueberry was constructed. The results of the joint analysis showed that the 228 functional genes and 42 TFs regulated 78 differentially expressed metabolites within the biosynthesis pathway of phenylpropanoids/flavonoids. O2PLS analysis results showed that the key metabolites differentially accumulated in blueberry fruit tissues were albireodelphin, delphinidin 3,5-diglucoside, delphinidin 3-O-rutinoside, and delphinidin 3-O-sophoroside, and 10 structural genes (4 *Vc4CLs*, 3 *VcBZ1s*, 1 *VcUGT75C1*, 1 *VcAT*, and 1 *VcUGAT*), 4 transporter genes (1 *VcGSTF* and 3 *VcMATEs*), and 10 TFs (1 *VcMYB*, 2 *VcbHLHs*, 4 *VcWD40s,* and 3 *VcNACs*) exhibited strong correlations with 4 delphinidin glycosides. These findings provide insights into the molecular mechanisms of flavonoid biosynthesis and accumulation in blueberry fruit.

## 1. Introduction

Blueberry (*Vaccinium* spp.) is a shrub plant belonging to the Ericaceae family. Based on their cold hardiness and chilling requirements for flowering, blueberry species can be further separated into lowbush blueberries (*V*. *angustifolium*), northern highbush blueberries (*V*. *corymbosum*), half-highbush blueberries (*V*. *corymbosum × V*. *angustifolium*), southern highbush blueberries (*V*. *australe*), and rabbiteye blueberries (*V*. *ashei*) [1]. In recent years, blueberry cultivation is almost ubiquitous worldwide due to the rising demand [2].

Blueberries are popular with consumers around the world because of their high nutritional value. The fruit has numerous beneficial effects for humans. Its active constituents can promote retinoid re-synthesis and improve immunity, as well as having anti-inflammatory, anti-cardiovascular disease, antiaging, and anticancer effects, which greatly promote human health [3]. Consumers consider blueberries “superfoods” because they contain high concentrations of antioxidants. Phytochemical studies have revealed that the antioxidant activity of blueberry fruit depends on the contents of the main phytochemical products, particularly flavonoids (anthocyanins, flavonols, and proanthocyanidins) and phenolic acids [4]. Anthocyanins are the pigments responsible for the blueberry’s color. The individual anthocyanin profile of blueberries is complex, comprising six anthocyanidins (delphinidin, pelargonidin, malvidin, cyanidin, petunidin, and peonidin) [5]. Anthocyanidins can be glycosylated with various sugars, mainly glucose, galactose, arabinose, or xylulose, and different combinations of the anthocyanidins and glycosylated forms are found in blueberry. 

Blueberriesy are also rich in flavonols, with a predominance of quercetin derivatives [6,7], and proanthocyanidins, formed by the polymerization of catechin and/or epicatechin units [8]. Phenolic acids are mainly represented by chlorogenic acid, which is generated by the esterification of caffeic acid with a quinic acid molecule [8,9]. The biosynthesis of flavonoids has been explored in various plants such as *Arabidopsis thaliana* [10], *Ananas comosus* [11], *Vitis vinifera* [12], *Citrus grandis* [13], and *Solanum tuberosum* [14]. The structural genes involved in the biosynthesis pathways of flavonoids can be divided into two groups: early biosynthesis genes (EBGs) and late biosynthesis genes (LBGs). The EBGs are highly conserved across the plant kingdom, including *Vaccinium* species, and mainly include structural genes encoding enzymes that catalyze the synthesis of naringenin from L-phenylalanine, such as phenylalanine ammonia lyase (PAL), cinnamate 4-hydroxylase (C4H), 4-coumarate-CoA ligase (4CL), chalcone synthase (CHS), and chalcone isomerase (CHI) [15,16]. The LBGs have not been fully explored in blueberry, and those characterized to date include genes encoding flavanone-3-hydroxylase (F3H), flavonol synthase (FLS), dihydroflavonol 4-reductase (DFR), anthocyanidin synthase (ANS), leucoanthocyanidin reductase (LAR), O-methyltransferase (OMT), acyltransferase (AT), and UDP Glc-flavonoid 3-O-glucosyltransferase (UFGT) [17]. Although some studies have focused on the synthesis and modification of anthocyanins in blueberry [5,17,18,19], there are still relatively few reports on the LBGs involved in anthocyanin modification and transport, including those genes involved in the glycosylation (glucosyl, galactosyl, arabinosyl, rutinosyl, and sophorosyl), acylation (caffeoyl, coumaroyl, and malonyl), and methylation of anthocyanins. These modifications contribute to the diversification and stabilization of anthocyanins. Studies have shown that some transcription factor genes (TFs) regulate anthocyanin biosynthesis and accumulation by binding to the promoter regions of structural genes related to anthocyanin synthesis [20,21,22]. For example, in *Arabidopsis thaliana*, anthocyanin biosynthesis can be controlled by the TTG1-bHLH-MYB (MBW) complex [23]. In addition, some other TFs can also regulate anthocyanin biosynthesis, such as WRKY [24], NAC [25], ERF [26], MADs-box [27], and bZIP [28].

The *Vaccinium corymbosum/angustifolium* cultivar Northland is a half-highbush blueberry with strong cold resistance, high yield, and good taste. It was produced from hybridize breeding *V. corymbosum* and *V. angustifolium*. Although high-quality genomes of southern highbush and northern highbush blueberry have been released, those of half-highbush blueberry have not yet been reported. Research on the biosynthesis of blueberry flavonoids is mainly at the single-omics level, and the mechanism of differential accumulation of flavonoids in the exocarp, sarcocarp, and seed of blueberry fruits is still unclear. In this study, the samples from mature fruits of Northland blueberry were sequenced by SMRT to obtain a more comprehensive and accurate full-length transcriptome sequence of half-highbush blueberry. Untargeted metabolome data were utilized to obtain a more comprehensive differentially expressed metabolite (DEM) profile, and Illumina sequencing technology was performed to obtain all transcriptome sequencing results for the three blueberry fruit tissues. The results of KEGG enrichment analysis showed that these DEMs and differentially expressed genes (DEGs) were significantly enriched in the biosynthesis pathway of flavonoids. Furthermore, a comprehensive analysis of transcriptome and metabolome more accurately screened out key genes regulating flavonoids in three different tissues of blueberry fruit. The research results clarified the molecular mechanism of flavonoid biosynthesis and accumulation in blueberry, especially the modification and transport of anthocyanins, and provided a basis for genetic improvement and germplasm innovation of blueberry.

## 2. Results

### 2.1. Untargeted Metabolomic Analysis: Differentially Expressed Metabolites in Blueberry Fruit Tissues 

**Figure 1:**
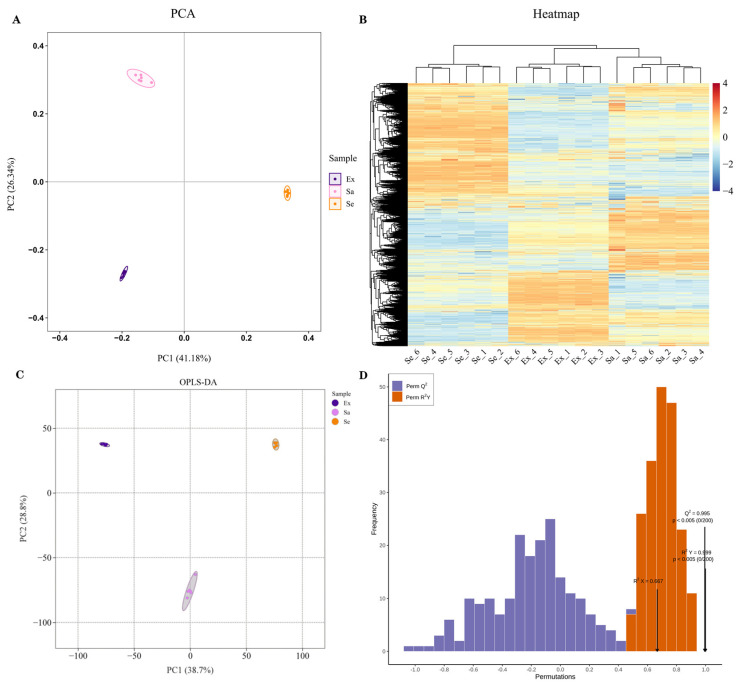
Metabolome analysis of mature blueberry fruit tissues; Ex, Sa, and Se. (**A**) A PCA score plot of metabolome data for the three sample groups. (**B**) An HCA heatmap of 18 samples. (**C**) An OPLS-DA score plot among three groups. (**D**) An OPLS-DA model verification diagram. R2X and R2Y represent the explanation rate of the X and Y matrices of the built model, respectively, and Q2 represents the predictive ability of the model (200 random permutation and combination experiments). An excellent model is achieved when Q2 > 0.9 and *P_R2Y_* < 0.05. The black arrows are used to indicate the values of the abscissa corresponding to each indicator.

To reveal the distribution of metabolites in blueberry fruit, an untargeted metabolome analysis was performed. The metabolic profiles of 18 samples, i.e., 6 replicates each of the exocarp (Ex), sarcocarp (Sa), and seed (Se) of mature blueberry fruit, were determined. A principal component analysis (PCA) showed that PC1 and PC2 accounted for 41.18% and 26.34% of the variation in metabolite accumulation, respectively. In the PCA score plot, the Ex, Sa, and Se sample groups were clearly separated, and the replicate samples were clustered compactly together (Figure 1A), thus indicating that the experimental data were reproducible and reliable. Hierarchical cluster analysis (HCA) of all detected features indicated significant differences in the relative abundance of metabolites among Ex, Sa, and Se (Figure 1B). Orthogonal partial least-squares discrimination analysis (OPLS-DA) is an effective method for discovering differentially expressed metabolites (DEMs) among samples as it maximizes differences between populations. In the plot of OPLS-DA scores, the R2Y score was 0.999 and Q2 was 0.995, demonstrating that these models were stable and reliable and could be used to further screen for DEMs (Figure 1C,D).

The DEMs between pairs of groups were identified on the basis of fold change (FC) and variable importance in project (VIP) values of the OPLS-DA model, where VIP ≥ 1, |Log_2_FC| ≥ 2, and a false discovery rate (FDR) < 0.01 were the criteria for differential expression. There were 424 DEMs between Ex and Sa (355 up-regulated; 69 down-regulated), 1159 between Ex and Se (595 up-regulated; 564 down-regulated), and 624 between Se and Sa (450 up-regulated; 174 down-regulated). 

### 2.2. Classification Statistics and KEGG Enrichment Analysis of DEMs

**Figure 2:**
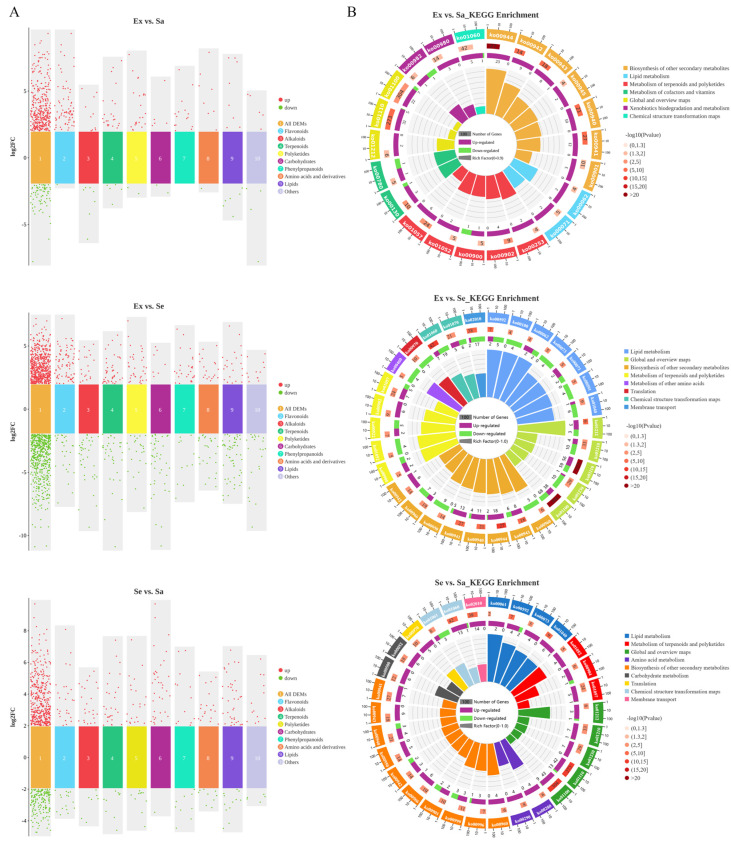
Classification statistics and KEGG enrichment analysis of DEMs in Ex vs.Sa, Ex vs. Se, and Se vs. Sa comparison groups. (**A**) Classification statistics of DEMs in comparison groups. (**B**) KEGG enrichment analysis of DEMs in three comparison groups.

In total, 835 DEMs were classified into 167 flavonoids, 64 phenylpropanoids, 82 alkaloids, 104 terpenoids, 83 polyketides, 78 carbohydrates, 70 amino acids and derivatives, 118 lipids, 30 glycosides, 17 nucleic acids, 14 organic acids, and 8 vitamins and cofactors. Among them, flavonoids were the largest group of DEMs and were mainly up-regulated in the exocarp compared with the sarcocarp and seed (Figure 2A, Table S1). 

The DEMs were significantly enriched in 56 KEGG pathways (*p* < 0.05, Figure 2B, Table S2), of which 12 KEGG pathways were commonly enriched in all three samples, namely, 6 secondary metabolite biosynthesis pathways [phenylpropanoid biosynthesis (ko00940), flavonoid biosynthesis (ko00941), anthocyanin biosynthesis (ko00942), isoflavonoid biosynthesis (ko00943), biosynthesis of plant secondary metabolites (ko01060), and biosynthesis of secondary metabolites (ko01110)] and 6 metabolism pathways [fatty acid elongation (ko00062), fatty acid degradation (ko00071), fatty acid metabolism (ko01212), type I polyketide structures (ko01052), and biosynthesis of type II polyketide products (ko01057)]. In the Ex vs. Sa comparison, the DEMs were mainly up-regulated in the exocarp. The DEMs included 48 flavonoids and 12 phenylpropanoids, which were enriched in 5 biosynthesis pathways associated with flavonoids and phenylpropanoids (*p* < 0.0001), and 4 monoterpenoids, which were enriched in the monoterpenoid biosynthesis pathway (*p* < 0.01). In the Ex vs. Se and Se vs. Sa comparisons, some DEMs were enriched in secondary metabolite pathways related to flavonoids, phenylpropanoids, and monoterpenoids, some were enriched in pathways of lipid biosynthesis, carbohydrate biosynthesis, and terpenoid metabolism, and some were enriched in the ABC transporter pathway (*p* < 0.0001).

A heatmap was drawn to illustrate the relative expression levels of 269 DEMs in the KEGG pathways (Figure S1). The results show that certain compounds were common among the DEMs in the three comparison groups, especially flavonoids and phenylpropanoids, including 26 flavones and flavonols, 13 anthocyanidins and anthocyanins, 7 isoflavones, 5 flavanols, 3 proanthocyanidins, 3 chalcones and dihydrochalcones, 3 flavanones, 3 dihydroflavonols, 3 pterocarpans, 2 isoflavanes, 1 aurone, 1 isoflavanone, 17 monolignols, 4 coumarin derivatives, and 1 lignan. These substances are related to flavor and fruit coloring, and were mainly highly expressed in the exocarp. The dark-purple or blue anthocyanins included albireodelphin, delphinidin 3,5-diglucoside, tulipanin, delphinidin 3-O-(6-caffeoyl-β-D-glucoside), delphinidin 3-O-sophoroside, delphinidin 3-O-glucoside, and delphinidin 3-glucoside 5-caffoyl-glucoside, and the orange or deep-red anthocyanins included cyanidin 3,5,3’-tri-O-glucoside, chrysanthemin, pelargonidin 3-O-(6-O-malonyl-β-D-glucoside), pelargonidin 3-O-β-d-glucoside 5-O-(6-coumaroyl-β-D-glucoside), and pelargonidin 5-O-β-D-glucoside 3-O-β-D-sambubioside. Naringenin, which has a bitter flavor, showed the highest relative abundance in seeds, while two flavanone glycosides (hesperetin 7-O-glucoside and naringenin 7-O-glucoside) showed the highest relative abundance in the exocarp. Terpenoids, which contribute to fruit aroma, were concentrated in the exocarp (seven monoterpenoids) and seed (seven polyterpenoids).

To verify the accuracy of the untargeted metabolome data, 30 anthocyanins were identified by Waters UHPLC tandem quadrupole (Figure S2). The results were consistent with the untargeted metabolome data and showed that anthocyanins were mainly highly expressed in the fruit exocarp. 

### 2.3. Full-Length Transcriptome Analysis of Mature Fruit

Considering that the half-highbush blueberry genome has not been published, PacBio was used to sequence the full-length transcriptome of mature fruits of the Northland cultivar. High-quality RNA samples from three different tissues (exocarp, sarcocarp, and seed) of blueberry mature fruits were sequenced, and nine Illumina sequencing libraries were constructed. A total of 9.4 Gb of data was obtained using the Pacbio RSII platform, with an average length of 1496 bp. After correction and clustering, 267,687 full-length non-chimeric (FLNC) reads with an average length of 2518 bp were obtained from the 390,855 circular consensus sequence (CCS) reads. To improve data accuracy, the third-generation sequences were further corrected with next-generation sequencing (NGS) data using LoRDEC software (v0.7). After correction, a total of 147,569 consensus transcripts were obtained with an average length of 2738 bp and an N50 of 3176 bp. Polished consensus sequences were aligned to the reference genome (*Vaccinium corymbosum* cv. Draper v1.0 genome sequence) using GMAP software (v2017-06-20). The alignment rate with the reference genome was 94.13%. Using TAPIS software (v1.2.1), the consensus sequences were further corrected and clustered, and redundancies were removed. This resulted in 63,425 final high-quality isoforms. Among them, 7584 were classified as isoforms of known genes, 49,005 as novel isoforms of known genes, and 6836 as isoforms of novel genes. Compared with the reference genome, the full-length transcriptome obtained here had longer transcript sequences (Figure S3), more new transcripts (Figure S3), and more transcript exons (Figure S3), indicating that this full-length transcriptome had a more complete and authentic structure. To obtain comprehensive annotation information, 5530 novel genes that were unmapped to the reference genome were annotated by comparison of their sequences with those in seven databases (NR, NT, Pfam, KOG/COG, SwissProt, KEGG, and GO) (Table 1, Figure S3). 

**Figure 3:**
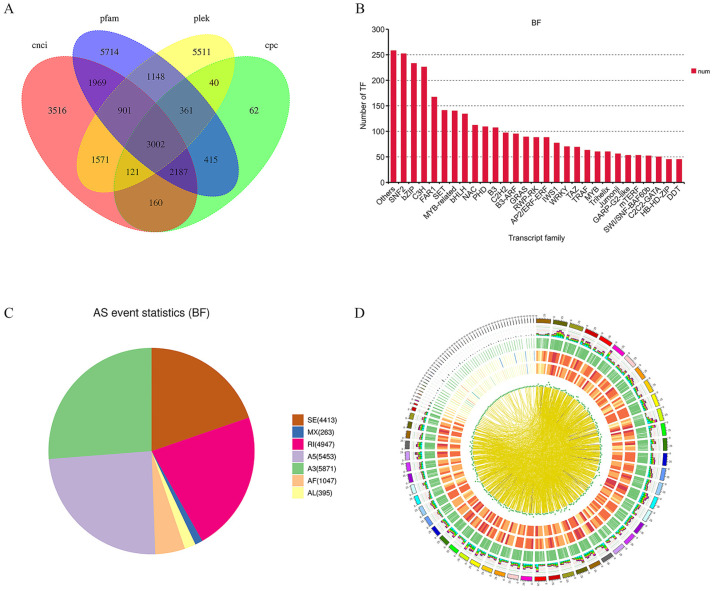
Characterization of non-redundant transcripts. (**A**) Venn diagram of lncRNAs predicted using four databases. (**B**) Number of genes encoding different transcription factors. (**C**) Statistics of seven types of alternative splicing events. (**D**) Diagram of genetic structure.

After filtering using four approaches (CPC, CNCI, PLEK, and Pfam), 3002 lncRNAs were finally obtained, of which 1663 lncRNAs belonged to 1496 novel genes (Figure 3A). In addition, a total of 3946 TF genes were identified from the PacBio Iso-Seq reads, encoding TFs in 89 categories. The top four TF families, in terms of the number of genes, were bZIP, MYB and MYB-related, bHLH, and NAC (Figure 3B). Alternative splicing (AS) is an important mechanism regulating gene expression and generating proteome diversity. A total of 30,540 AS events corresponding to 12,607 gene loci were identified. These AS events were divided into categories of alternative 3′ splice-site (A3, 19.22%), alternative 5′ splice-site (A5, 17.86%), retained intron (RI, 16.20%), skipped exon (SE, 14.45%), alternative first exon (AF, 3.43%), alternative last exon (AL, 1.29%), and mutually exclusive exon (MX, 0.86%). Full-length transcripts were also used to identify transcript fusion events (Figure 3C). A total of 985 fusion events were found, consisting of 14 inter-chromosomal and 971 intra-chromosomal fusion transcripts (Figure 3D). 

### 2.4. Identification and Functional Enrichment Analysis of DEGs

**Figure 4:**
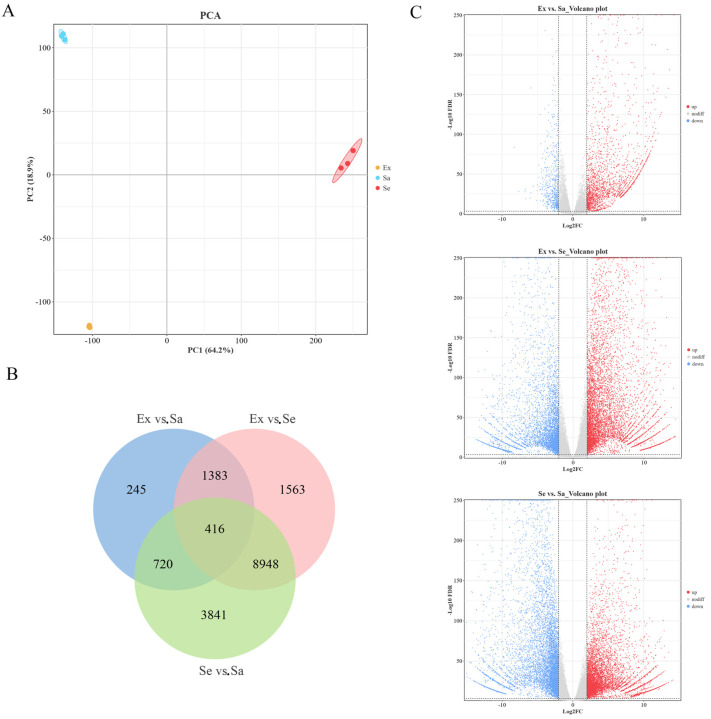
Transcriptome analysis of different Northland blueberry fruit tissues. (**A**) Principal component analysis (PCA) plot of DEGs in exocarp, sarcocarp, and seed of blueberry. (**B**) Venn diagram showing DEGs among three sample groups. (**C**) Volcano plot showing DEGs in each pairwise comparison.

The transcriptome profiling of nine blueberry samples was expected to provide useful data for further studies on the molecular basis of the metabolic differences among the exocarp, sarcocarp, and seed. The majority (87.06~89.08%) of clean reads were successfully mapped to the reference genome (Table S3). The results indicated that the sequencing results were of good quality and could be used for further analyses. A PCA was conducted using the transcriptome data from the nine blueberry samples. The results showed that PC1 and PC2 explained 64.2% and 18.9% of the total variance among samples, respectively. The biological replicates of each group clustered together, and the three groups were clearly separated (Figure 4A). The DEGs were detected in pairwise comparisons (Ex vs. Sa, Ex vs. Se, and Se vs. Sa) according to the criteria of |Log_2_FC| ≥ 2 and FDR < 0.001. A total of 2764 (2158 up-regulated and 606 down-regulated), 12,310 (7159 up-regulated and 5151 down-regulated), and 13,925 (6611 up-regulated and 7314 down-regulated) DEGs were obtained in the pairwise comparisons of Ex vs. Sa, Ex vs. Se, and Se vs. Sa, respectively, with 416 DEGs common to the three tissues (Figure 4B,C). 

**Figure 5:**
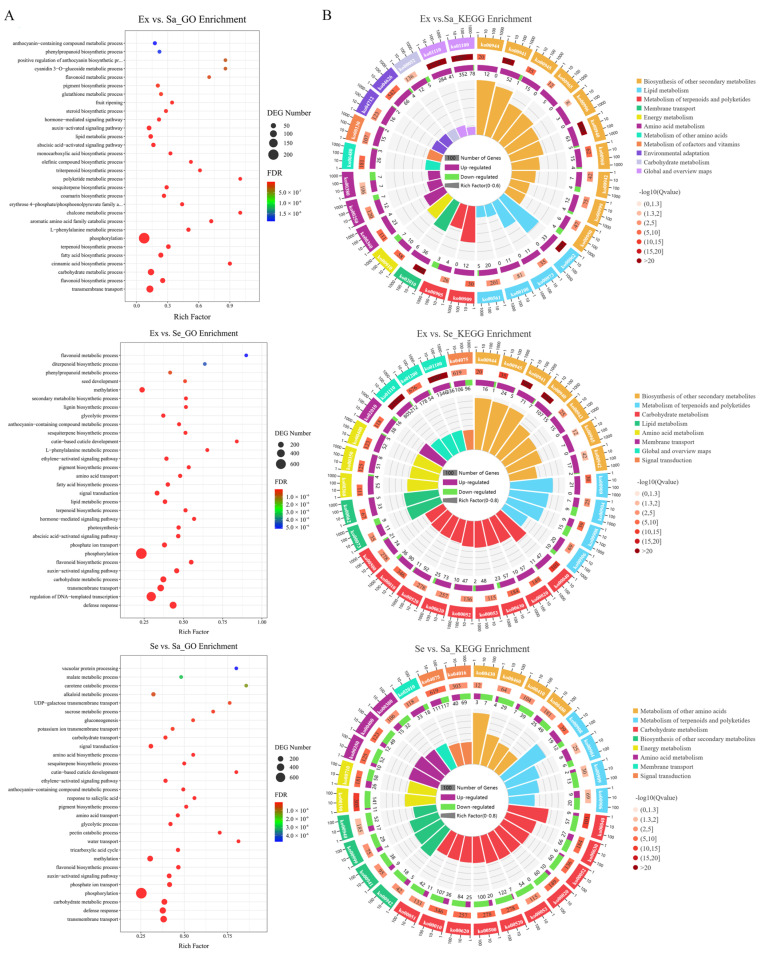
Functional enrichment analysis of DEGs. (**A**) GO enrichment analysis plot. (**B**) KEGG enrichment analysis plot.

A GO functional enrichment analysis of DEGs was conducted (FDR < 0.05, Figure 5A, Table S4). The DEGs were classified into three main functional GO categories, cellular component, biological process, and molecular function, as the first step in establishing their biological functions. There were 219 commonly significantly enriched GO terms in the three comparison groups. In the cellular component category, the most highly represented GO terms were membrane, extracellular region, and cell periphery, followed by vacuole, apoplast, and cytoplasm in the three combination groups. Nucleus, chloroplast, and mitochondrion were only highly represented in the comparison groups containing seed samples. In the molecular function category, the most highly represented GO terms in the three comparison groups were catalytic activity, binding, and transporter activity, which mainly consisted of oxidoreductase activity, carbohydrate binding, transmembrane transporter activity, signaling receptor activity, and transferase activity. In the biological process category, the highly represented GO terms were mainly related to the biosynthesis of primary metabolites and secondary metabolites and the transmembrane transport process, such as transmembrane transport, the flavonoid biosynthetic process, the carbohydrate metabolic process, the sesquiterpene biosynthetic process, the auxin-activated signaling pathway, the pigment biosynthetic process, and the anthocyanin-containing compound metabolic process (Figure 5A). In total, 187, 249, 548, 136, 144, and 431 DEGs were enriched in GO entries related to flavonoids, phenylpropanoids, carbohydrates, terpenoids, alkaloids, and lipids, respectively (Table S5).

Next, KEGG pathway enrichment analyses were performed for the DEGs (FDR < 0.05, Table S6) detected in the three different comparisons: Ex vs. Sa, Ex vs. Se, and Se vs. Sa (Figure 5B). The DEGs detected in the Ex_vs_Sa comparison were enriched in 29 KEGG pathways, and 157 DEGs (139 up-regulated and 18 down-regulated) were significantly enriched in 10 secondary metabolite biosynthesis pathways, including flavonoid biosynthesis (ko00941), phenylpropanoid biosynthesis (ko00940), flavone and flavonol biosynthesis (ko00944), and anthocyanin biosynthesis (ko00942). For the DEGs detected in the Ex vs. Se comparison, phenylpropanoid biosynthesis and flavonoid biosynthesis were the most enriched pathways, followed by stilbenoid, diarylheptanoid, and gingerol biosynthesis (ko00945), and pentose and glucuronate interconversions (ko00040), which contained 122, 78, 29, and 58 genes, respectively. For the DEGs detected in the Se vs. Sa comparison, the most enriched pathways were pentose and glucuronate interconversion, amino sugar and nucleotide sugar metabolism (ko00520), and zeatin biosynthesis (ko00908), and the pathways with the most genes were carbon metabolism (ko01200), plant hormone signal transduction (ko04075), and the biosynthesis of amino acids (ko01230), which contained 262, 228, and 209 genes, respectively. Overall, most DEGs were related to phenylpropanoids, flavonoids, carbohydrates, terpenoids, amino acids, and transporters and signal transduction, all of which may be involved in flavor compound synthesis. In addition, genes related to flavonoid biosynthesis were among the DEGs in all of the comparison groups. These results were basically consistent with those of the metabolomic analysis.

### 2.5. Integrated Transcriptome and Metabolome Analysis

Through a joint analysis of metabolomics and transcriptomics, DEGs and DEMs that were significantly enriched and strongly correlated in the flavonoid biosynthesis pathways were characterized. In addition, the correlations between DEGs and DEMs were determined based on Pearson correlation’s coefficient (|Cor| ≥ 0.95, *p* < 0.001). To further verify the accuracy of the annotation information, the KEGG hmmer database (v2023-12-30) and Web-CDD online software (v3.17) were used to check sequence integrity and conserved domains.

**Figure 6:**
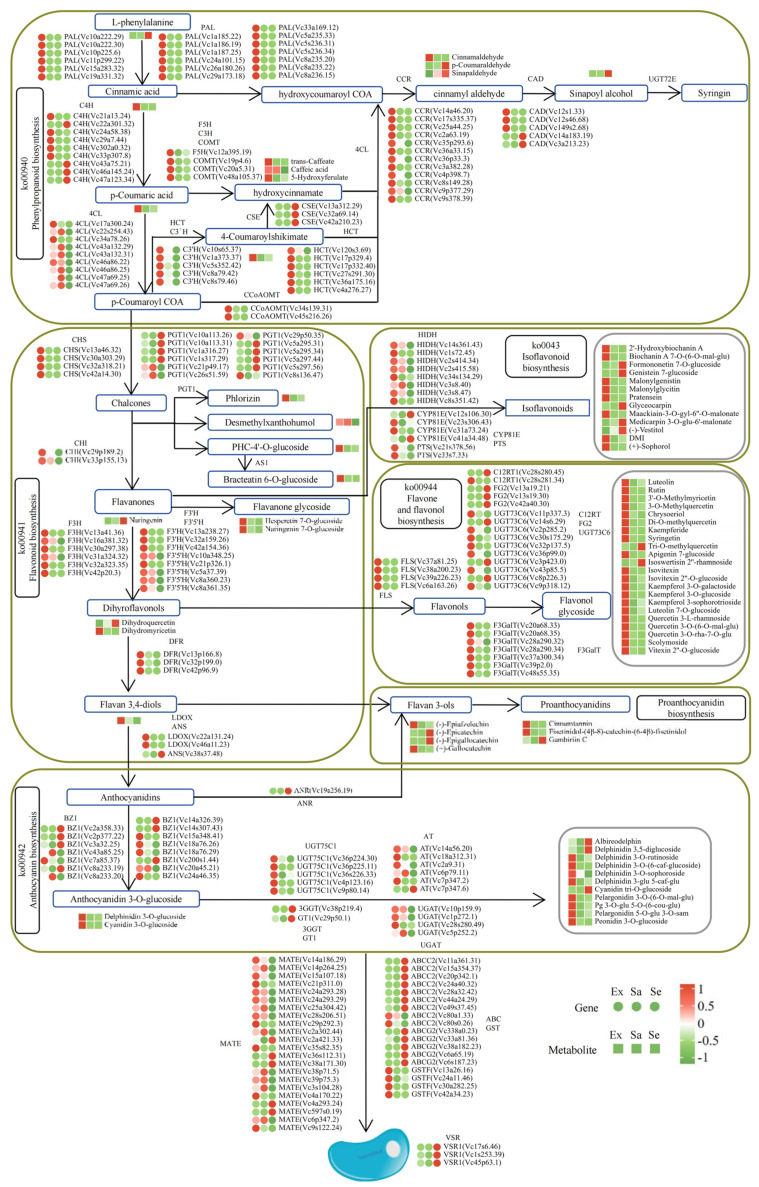
Flavonoids’ metabolism pathway maps, including metabolite and structural gene expression in Ex, Sa, and Se of blueberry. For each gene/metabolite, left, middle, and right circle or square indicate relative expression level in Ex, Sa, and Se, respectively.

Flavonoids are widely distributed in plants and have attracted much attention because of their functions in physiological activities. To further explore the biosynthesis of flavonoids in blueberry, the genes and metabolites involved in phenylpropanoid and flavonoid biosynthesis (ko00940, ko00941, ko00942, ko00943, and ko00944) were identified based on the enriched KEGG pathways (Figure 6). The phenylpropanoid biosynthesis pathway leads to the synthesis of phenolic acids, lignin, and monolignols. In total, 74 DEGs encoding 11 key enzymes were involved in the biosynthesis of phenylpropanoids (Table S7), including 19 genes encoding PAL, 9 encoding C4H, 9 encoding 4CL, 5 encoding 5-O-(4-coumaroyl)-D-quinate 3′-monooxygenase (C3′H), 3 encoding caffeic acid 3-O-methyltransferase/acetylserotonin O-methyltransferase (COMT), 1 encoding ferulate-5-hydroxylase (F5H), 12 encoding cinnamoyl-CoA reductase (CCR), 5 encoding cinnamyl-alcohol dehydrogenase (CAD), 6 encoding shikimate O-hydroxycinnamoyltransferase (HCT), 3 encoding caffeoylshikimate esterase (CSE), and 2 encoding caffeoyl-CoA O-methyltransferase (CCoAOMT). Flavonoid biosynthesis requires biosynthetic and modification enzymes and regulatory factors that control the expression of structural genes. Transporters are involved in flavonoid transport and storage. PAL, 4CL, and C4H encode upstream structural enzymes in flavonoid biosynthesis. Another 79 DEGs encoding enzymes involved in flavonoid synthesis and modification were also identified, including four genes encoding CHS, two encoding CHI, six encoding F3H, three encoding flavonoid 3′-monooxygenase (F3′H), five encoding flavonoid 3′,5′-hydroxylase (F3′5′H), four encoding FLS, three encoding flavonol 3-O-glucoside L-rhamnosyltransferase (FG2), seven encoding flavonol 3-O-β-D-galactosyltransferase (F3GalT), ten encoding flavonol 3-O-L-rhamnoside-7-O-glucosyltransferase (UGT73C6), three encoding DFR, one encoding ANR, one encoding ANS, two encoding leucoanthocyanidin dioxygenase (LDOX), eight encoding 2-hydroxyisoflavanone dehydratase (HIDH), twelve encoding phlorizin synthase (PGT1), two encoding flavanone 7-O-glucoside 2″-O-beta-L-rhamnosyltransferase (C12RT1), four encoding isoflavone/4′-methoxyisoflavone 2′-hydroxylase (CYP81E), and two encoding pterocarpan synthase (PTS). Among them, FLS, FG2, C12RT1, F3GalT, and UGT73C6 are involved in the biosynthesis of flavones and flavonols, CYP81E, HIDH, and PTS are involved in the biosynthesis of isoflavonoids, and DFR, ANS, and ANR are involved in the biosynthesis of anthocyanidins and proanthocyanidins. The anthocyanin biosynthesis pathway, which is downstream of flavonoid biosynthesis, is responsible for the methylation, glycosylation, or acylation of anthocyandins to form stable anthocyandin glycosides, which are stored in the vacuole. The DEGs in the anthocyanin pathway included fifteen encoding anthocyanidin 3-O-glucosyltransferase (BZ1), one encoding anthocyanidin 5,3-O-glucosyltransferase (GT1), one encoding anthocyanidin 3-O-glucoside 2″-O-glucosyltransferase (3GGT), five encoding anthocyanidin 3-O-glucoside 5-O-glucosyltransferase (UGT75C1), six encoding AT, and four encoding cyanidin 3-O-glucoside 2″-O-glucuronosyltransferase (UGAT). 

Anthocyanins are synthesized in the cytoplasm and stored in the vacuole. Three main transporter families were investigated: glutathione-S-transferase (GST), multidrug and toxic compound extrusion (MATE) transporter, and ATP-binding cassette (ABC) transporter. The results of the Pearson’s correlation coefficient analyses (Cor ≥ 0.95, *p* < 0.001) indicated that 40 DEGs encode proteins that may be involved in anthocyanin transport, including 4 GSTF family members, 22 MATE family members, 9 ABCCs (MRP), and 5 ABCG family members. These DEGs encoded transporters that may be involved in the transport and storage of phenylpropanoids and flavonoids in blueberry. In addition, Vacuolar Sorting Receptor (VSR) protein is a membrane protein in plants. It is responsible for participating in the directional transport and breakdown of vacuoles in plant cells, and three VSRs were characterized.

In the anthocyanin biosynthesis pathway, multiple families of TFs are involved in the regulation of biological processes that occur at different stages. Binding sites for six TF families were identified in the 2000 bp region upstream of the CDS sequences of all candidate genes using the online software PlantPAN 4.0. The six TFs with binding sites in the gene promoter regions were MYB, bHLH, WRKY, MADS-box, NAC, and bZIP. However, no binding sites of WD40 family members, which are related to anthocyanin biosynthesis, were found in the promoter regions. Hmmer searches and Web-CDD analyses verified that 2345 of the DEGs encoded TFs (DEFs), including 51 MYBs, 34 bHLHs, 14 WD40s, 44 WRKYs, 35 NACs, 29 MADS-boxes, and 35 bZIP TFs. These TF families have all been reported to be involved in anthocyanin biosynthesis. The results of Pearson’s correlation coefficient analyses identified 11 bHLHs, 7 MYBs, 6 WD40s, 4 bZIPs, 10 NACs, and 4 WRKYs as putative TFs involved in flavonoid biosynthesis in blueberry (Table S8).

### 2.6. Candidate Hub Genes and Metabolites Related to Flavonoid Biosynthesis

**Figure 7:**
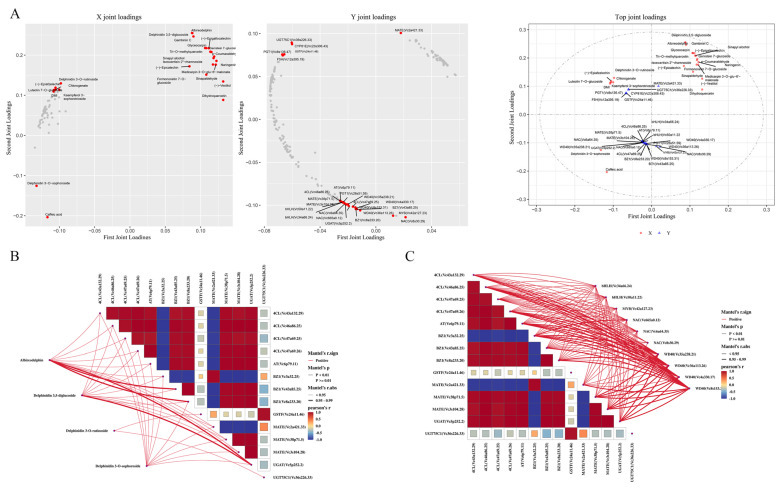
(**A**) O2PLS analysis. (**B**) A correlation network heatmap showing the connections between DEMs and DEGs enriched in the flavonoid biosynthesis pathway. (**C**) A correlation network heatmap showing the connections between DEFs and DEGs enriched in the flavonoid biosynthesis pathway. The thicker the line, the higher the correlation coefficient.

We conducted an O2PLS analysis to mine the major genes and metabolites associated with the transcriptome and metabolome in blueberry fruit. To determine which metabolites and genes were related to each other, we constructed two omics association loading plots for some of the related variables (genes and metabolites) (Figure 7). The results showed that the DEMs with the highest correlations were anthocyanins, and the top 20 metabolites included 4 anthocyanin glycosides, all of which were delphinidin glycosides (albireodelphin, delphinidin 3,5-diglucoside, delphinidin 3-O-sophoroside, and delphinidin 3-O-rutinoside). The top 20 DEGs included 4 encoding 4CL (*Vc43a132.29*, *Vc46a86.25*, *Vc47a69.25*, and *Vc47a69.26*), 1 encoding AT (*Vc6p79.11*), 3 encoding BZ1 (*Vc3a32.25*, *Vc43a85.25*, and *Vc8a233.20*), 1 encoding GSTF (*Vc24a11.46*), 3 encoding MATE (*Vc2a421.33*, *Vc38p71.5*, and *Vc3s104.28*), 1 encoding UGAT (*Vc5p252.2*), and 1 encoding UGT75C1 (*Vc36s226.33*). Ten DEGs encoding TFs were also among the hub genes, including two encoding bHLH (*Vc34a66.24* and *Vc50a11.22*), one encoding MYB (*Vc42a127.23*), three encoding NAC (*Vc665a0.12*, *Vc6a64.35*, and *Vc8s30.29*), and four encoding WD40 (*Vc35a238.21*, *Vc36a113.26*, *Vc4a330.17*, and *Vc8s153.31*). Other core metabolites were three proanthocyanidins (epicatechin, epigallocatechin, and gambiriin C), one flavanone (naringenin), three flavonols (dihydroquercetin, tri-O-methylquercetin, and isoswertisin 2″-rhamnoside), five isoflavonoids (formononetin 7-O-glucoside, genistein 7-glucoside, vestitol, glyceocarpin, and medicarpin 3-O-glu-6′-malonate), and four phenylpropanoids (p-coumaraldehyde, caffeic acid, sinapaldehyde, and sinapyl alcohol). These are interesting antioxidant and/or flavor substances that wait further verification.

### 2.7. Quantitative Real-Time PCR Analysis

**Figure 8:**
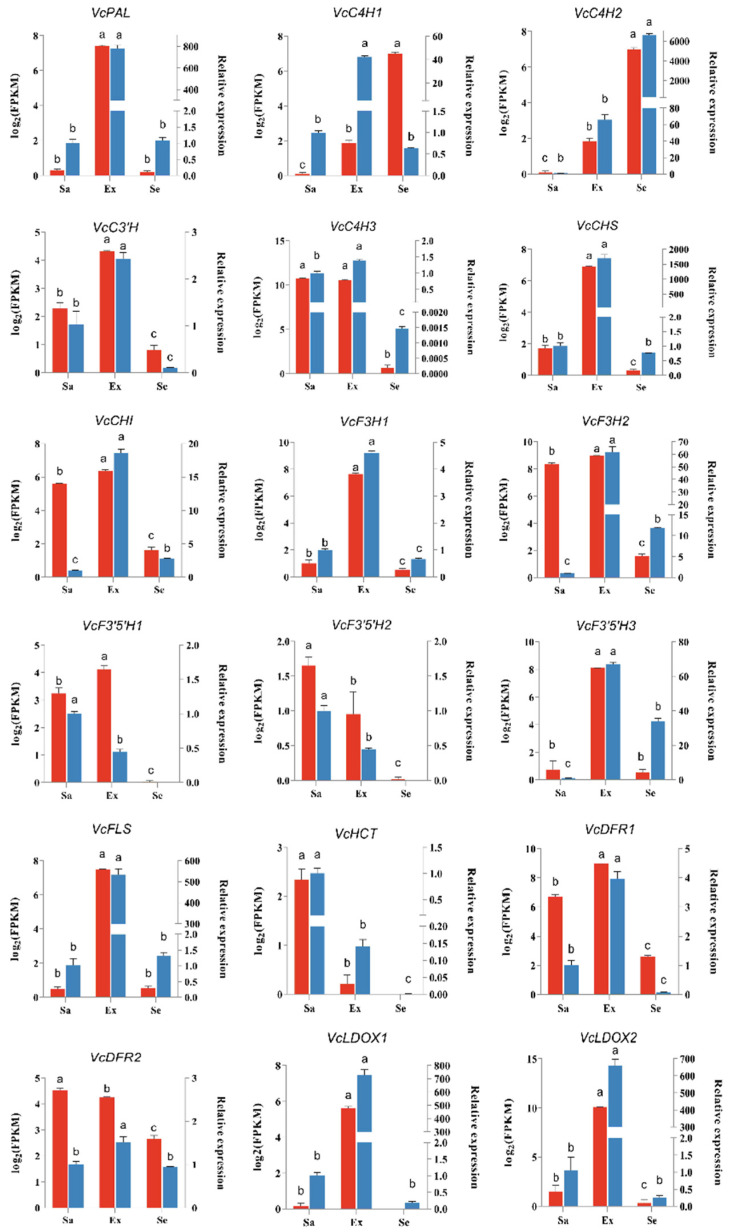
qRT-PCR validation of 18 DEGs in blueberry fruit tissues: Ex, Sa, and Se. The Y-axis on the left represents the gene transcript levels calculated as Log_2_FPKM, shown in red; the Y-axis on the right represents the relative expression levels (2^−∆∆Ct^) analyzed by qRT-PCR, shown in blue. Error bars indicate standard errors (*n* = 3). Small letters (a, b, and c) represent a statistically significant difference (*p* < 0.05) analyzed using Student’s *t*-test.

To validate the accuracy of the RNA-seq data, we randomly selected 18 genes involved in the flavonoid biosynthesis pathway for quantitative real-time PCR (qRT-PCR) analysis. As detected by qRT-PCR, the transcript levels of most genes were higher in the exocarp, consistent with the pattern of anthocyanin accumulation in the fruit. The results validated those obtained from the RNA-Seq data. The transcript profiles of both up-regulated and down-regulated genes were consistent between the qRT-PCR and RNA-Seq results (Figure 8).

## 3. Discussion

The first full-length (FL) transcriptome and nine Illumina sequencing libraries of half-highbush blueberry mature fruits were generated using the Northland cultivar, with samples from three fruit tissues: exocarp, sarcocarp, and seed. The FL transcriptome of blueberry fruit consisted of 147,569 transcripts, 94.13% of which were matched to the reference genome sequence. There were 63,425 high-quality FL isoforms in Northland, with an average length of 2679 bp and an N50 of 3112 bp, higher than the 32,119 and 31,323 FL transcripts reported for southern highbush blueberry cultivars Suziblue and Powderblue, respectively, with a maximum average length of 1770 bp [5]. These results confirm that the sequencing results in this study are of good quality. Full-length transcriptome data allow for the discovery new functional genes and for accurate analyses of structural information, such as alternative splicing variants and fusion genes of reference genome species [29]. In this study, 4034 new functional genes and 1496 new lncRNAs were discovered. These data supplement existing genome annotation information. More than half (59.84%) of non-redundant sequences had no other isoforms, whereas 40.16% of the sequences had more than one isoform, indicating that alternative splicing events are common. In this study, although RI (16.20%) and SE (14.45%) were among the main alternative splicing events, A3 (19.22%) and A5 (17.86%) also accounted for higher proportions, which differs from the case in other blueberry cultivars [30]. This may be related to the fruit development period or fruit tissues used in this study. In strawberry, intron retention is the most abundant alternative splicing event, but during fruit development, the proportion changes and selective acceptor sites become the main alternative splicing event after fertilization. It has also been suggested that splicing patterns in blueberry change depending on the fruit development stage. The abundance of A5 and A3 have important implications for gene regulation and protein diversity [31]. Other researches have highlighted alternative splicing as a potential approach to modulate anthocyanin biosynthesis in specific tissues; for example, CmbHLH2^Full^ (encoding the complete HLH2 TF) and its interaction with CmMYB6 were found to play key roles in the changes in anthocyanin pigmentation in ray florets in chrysanthemum, whereas CmbHLH2^Short^ (encoding a truncated HLH2) did not [32]. In this study, 47 DEGs encoding TFs related to flavonoids had alternative splicing variants, although further research is required to determine how they exert their regulatory effects. Together, these results indicate that alternative splicing events effectively broaden the length and number of single blueberry genes and provide valuable resources for future molecular biology research on blueberry. Although there is also increasing evidence for the health benefits of other *Vaccinium* species, including bilberry and lingonberry, much of this research has focused on blueberry and cranberry [33]. In this study, the DEMs among the Ex, Sa, and Se samples included 167 flavonoids, which were mainly accumulated in Ex samples. Flavonols such as quercetin, kaempferol, and isorhamnetin have been identified as stronger antioxidants than vitamin C compounds [34], and quercetin accounts for the largest proportion (75%) of total flavonols. In this study, quercetin was the most abundant flavonol in the three fruit tissues, followed by myricetin and kaempferol. The top three most abundant single substances in all samples were syringetin, 3-O-methylquercetin, and 3,7-di-O methylquercetin. Notably, methylation was the main modification of flavonols. In general, methylated flavonoids exhibit better physiological properties than non-methylated flavonoids, including greatly enhanced metabolic stability and increased membrane transport [35]. Anthocyanins are thought to be responsible for the health benefits of blueberries [8] and are also the main metabolites responsible for blueberry fruit coloration. In this study, thirteen anthocyanins were detected, namely, seven delphinins, three pelargonins, two cyanidins, and one peonidin. Delphinidins were the predominant anthocyanins in Northland blueberry fruit, consistent with previous reports on blueberry fruits at different developmental stages [36]. Our findings indicate that compounds contributing to flavor and aroma primarily accumulate in the exocarp and sarcocarp, such as flavonoids, terpenoids, monosaccharides, and organic acids. Oligosaccharides, polyterpenes, and lipids are predominantly stored in seeds, where they play roles in energy storage and embryonic development. Blueberry anthocyanins are mainly formed by an anthocyanin binding to a monosaccharide, so sugars can be used as raw materials to promote anthocyanin synthesis. It has been reported that there is a highly significant linear correlation between the soluble sugar content and anthocyanin content in fruits [37]. The accumulation of monosaccharides in the exocarp may promote the synthesis of anthocyanins, causing the color of the blueberry exocarp to deepen.

Flavonoid biosynthesis encompasses the biosynthesis pathways of phenylpropanoids, flavonoid, anthocyanins, isoflavonoids, flavones, and flavonols, which involve structural enzymes, modification enzymes, transporters, and regulatory factors [38]. Joint transcriptomics and metabolomics analyses provide a powerful approach to identify genes involved in flavonoid synthesis. This study used combined omics analysis to elucidate the molecular mechanisms underlying the biosynthesis of flavonoids and their differential accumulation in the blueberry exocarp, sarcocarp, and seed. The flavonoid-related structural genes detected in Northland blueberry were PAL, 4CL, C4H, CHS, CHI, FLS, F3H, F3′H, F3′5′H, DFR, LDOX, ANR, ANS, UFGT, etc. The anthocyanin biosynthesis pathway is downstream of flavonoid biosynthesis. The chemical structure of anthocyanidins is inherently unstable, so they are readily altered within plant cells. The more stable forms, anthocyanin aglycones, are synthesized via several chemical reactions, such as hydroxylation, glycosylation, methylation, and acylation [39,40,41,42]. Our O2PLS analysis results showed that four delphinidin glycosides, namely, albireodelphin, delphinidin 3,5-diglucoside, delphinidin 3-O-rutinoside, and delphinidin 3-O-sophoroside, were key metabolites differentially enriched among the three fruit tissues. All of these pigments are displayed as a dark-purple or blue color. Anthocyanins are synthesized at the external surface of the endoplasmic reticulum and stored inside the vacuole [43]. In this study, the transcript levels of 10 genes encoding structural and modification enzymes (4 *Vc4CLs*, 3 *VcBZ1s*, 1 *VcUGT75C1*, 1 *VcAT*, and 1 *VcUGAT*) and 4 transporter genes (1 *VcGSTF* and 3 *VcMATE*) were positively correlated with the relative abundance of anthocyanins, indicating that their encoded proteins are involved in anthocyanin biosynthesis and transport in Northland blueberry. Currently, the transport of anthocyanins is still poorly understood and requires additional research [33]. These genes may be important for the stable, deep coloration of the exocarp. The results also indicate that glycosylated and acylated anthocyanins play important roles in mature blueberry fruit. It is worth mentioning that the 15 anthocyanins found in highbush blueberry fruits are 3-O-glycosides of 5 anthocyanins, and no acetylated anthocyanins were detected [44]. Among the 25 anthocyanins detected in lowbush bilberries, including 14 anthocyanins except peonidin-arabinoside, 11 acetylated anthocyanins were also detected [45]. Simeone et al. found that in cold areas, blueberry may have a high acetylated anthocyanin content, and Yang et al. speculated that the production of acetylated anthocyanins is a protective mechanism of plants used to maintain a stable fruit color [5]. Ten acetylated anthocyanins were identified in Northland blueberry, which may be related to its complex genetic background and cold cultivation conditions. In addition, the following anthocyanins have not been identified yet in blueberry, namely, albireodelphin, delphinidin 3-O-(6-caffeoyl-glucoside), delphinidin 3-O-glucoside 5-O-(6-coumaroylglucoside), pelargonidin 3-O-(6-O-malonylglucoside), and pelargonidin 3-O-glucoside 5-O-(6-coumaroylglucoside), and the regulatory network of the key metabolite albireodelphin can enrich the theoretical basis for anthocyanin modification.

Transcription factors regulate the transcription and activity of structural genes involved in anthocyanin synthesis and accumulation. Analyses of the promoters of genes related to anthocyanin synthesis revealed binding sites for TFs in six families, i.e., MYB, bHLH, WRKY, MADS-box, NAC, and bZIP. It is worth noting that the promoter of each candidate gene contains binding sites for these six TFs, indicating that anthocyanin synthesis may be regulated by multiple TFs simultaneously. The MBW complex is known to directly regulate the transcription of flavonoid structural genes, but no WD40 family members related to anthocyanin biosynthesis were identified among the structural genes. The WD40 protein is not directly involved in specific recognition of target gene promoters, but has a more active role in enhancing gene activation [46]. It interacts with MYB and bHLH-like transcription factors to form the MBW ternary complex that regulates anthocyanin synthesis, which affects anthocyanin accumulation by activating the expression of structural genes in the anthocyanin synthesis pathway [47]. In *Arabidopsis*, *AtTT2*, *AtTT8,* and *AtTTG1* form the MBW complex to regulate anthocyanin synthesis. In this study, *Vc43p33.1* (*AtTT8*) and *Vc44s239.19* (*AtTT2*), which are homologs of the above genes, were found to possibly encode TFs with important roles in regulating the anthocyanin synthesis pathway. Overall, the results of this study shed light on the molecular mechanism of flavonoid synthesis and accumulation in blueberry fruit.

## 4. Materials and Methods

### 4.1. Plant Materials

Seven-year-old plants of the V. corymbosum/angustifolium cultivar Northland grow in the Small Berry Genetic Breeding Center of Jilin Agricultural University in Changchun, China (43°79′N, 125°41′E). The altitude is 259 m, the soil is sandy loam improved by sulfur powder and commercial peat soil, the organic matter content is 3.72%, pH value is 5.5–5.8. Mature blueberry fruits were harvested when the exocarp turn completely blue, the exo-carp (Ex), sarcocarp (Sa) and seed (Se) were picked with tweezer on ice. All samples were immediately frozen in liquid nitrogen, and then stored at −80 °C until analysis.

### 4.2. Metabolite Sequencing and Analysis

Six biological replicates of the Ex, Sa, and Se samples from mature blueberry fruits were analyzed. Firstly, the collected samples were thawed on ice, and metabolites were extracted using a 50% methanol buffer. In brief, 20 μL of each sample was mixed with 120 μL of precooled 50% methanol, vortexed for 1 min, and then incubated at room temperature for 10 min. The extraction mixture was stored overnight at −20 °C. After centrifugation at 4000× *g* for 20 min, the supernatant was transferred into a new 96-well plate. Additionally, pooled QC samples were prepared by combining 10 μL aliquots of all extraction mixtures. Secondly, all samples were acquired by the LC-MS system followed machine orders. All chromatographic separations were performed using an ultra-performance liquid chromatography (UPLC) system (SCIEX, UK). An ACQUITY-UPLC-T3 column (100 mm × 2.1 mm, 1.8 µm, Waters, UK) was used for the reversed phase separation. The column oven was maintained at 35 °C. The flow rate was 0.4 mL/min and the mobile phase consisted of solvent A (water, 0.1% formic acid) and solvent B (Acetonitrile, 0.1% formic acid). Thirdly, a high-resolution tandem mass spectrometer TripleTOF5600plus (SCIEX, UK) was used to detect metabolites eluted from the column. Finally, the MS data were subjected to pretreatments, including peak picking, peak grouping, retention time correction, second peak grouping, and annotation of isotopes and adducts, using XCMS software (v3.7.1). PCA and OPLS-DA analyses were conducted to confirm metabolic differences and reliability. With |Log_2_FC| ≥ 2, *p* < 0.05, and VIP ≥ 1 as thresholds, differentially expressed metabolites (DEMs) were screened. Subsequently, the DEMs were mapped to the KEGG database, and their significance was determined by a hypergeometric test *p*-value (*p* < 0.05).

### 4.3. PacBio Iso-Seq and RNA-Seq Library Construction and DEG Analysis

The total RNA of the samples was extracted using the modified CTAB method and its integrity was analyzed using an Agilent 2100 bioanalyzer (Agilent, Palo Alto, CA, USA). The Iso-Seq library of mature fruits was prepared according to the Isoform Sequencing protocol (Iso-Seq) using the Clontech SMARTer PCR cDNA Synthesis Kit and the BluePippin Size Selection System protocol, as specified by Pacific Biosciences (PN 100-092-800-03). The full-length library of the mature fruits was sequenced on the PacBio Sequel II platform in three SMRT cells. Full cDNA sequences were processed using SMRTlink 5.0 software and corrected with NGS data using LoRDEC software (v0.7) to obtain consensus unigenes. Redundant sequences (those with >99% similarity) were removed from the corrected transcript sequences using CD-HIT software (v4.8.1). The distribution of length frequency before and after removing redundant transcripts was counted.

The total RNA was extracted from Ex, Sa, and Se samples from mature blueberry fruit using the modified CTAB method. Transcriptome sequencing was performed on the Hiseq 2000 Illumina platform (Novogene Bioinformatics Technology Co. Ltd., Tianjin, China). Gene expression and transcript levels were calculated using RSEM (v1.2.8) software. According to the read count values of each sample, DEGs were identified in pairwise comparisons between groups using DESeq2 software (v1.42.1) with |Log2FC| ≥ 2 and FDR < 0.001 as the threshold. The DEGs in each sample were analyzed and subjected to GO functional annotation and KEGG enrichment analysis. 

### 4.4. Gene Functional Annotation 

Using GMAP, the third-generation consensus reads were aligned to the reference genome. The genome data of the *Vaccinium corymbosum* cv. Draper v1.0 were downloaded from https://www.vaccinium.org/genomes (accessed on 20 September 2023). Novel genes were annotated in the following databases: NR (NCBI non-redundant protein sequences), NT (NCBI non-redundant nucleotide sequences), Pfam (Protein family), KOG/COG (Clusters of Orthologous Groups of proteins), Swiss-Prot (a manually annotated and reviewed protein sequence database), KO (KEGG Ortholog database), and GO (pannzer2 database) [48,49]. TFs were predicted using the plantTF database from https://planttfdb.gao-lab.org (accessed on 15 December 2023) and online software PlantPAN 4.0 [50,51].

### 4.5. Integrated Metabolomic and Transcriptomic Analyses

Correlations between DEG transcript levels and DEM profiles were analyzed using the program R [52]. The FPKM values for genes and the peak values of metabolites were used as a matrix for Pearson’s partial correlation analysis [53]. Through O2PLS analysis, the internal connection between the two omics data was mined, the degree of correlation between the two omics data was determined, and the hub genes and metabolites that cause this correlation were determined [54,55].

### 4.6. qRT-PCR Analysis

Applied Biosystems (ABI) Primer Express software (v3.0.1) was used to design the qRT-PCR primers, and the primers are listed in Table S9. *EF1-α* was used as the internal reference gene [56]. Total RNA was converted to cDNA using the PrimeScript™ RT reagent Kit with gDNA Eraser (TaKaRa, Dalian, China). qRT-PCR was performed using the TB Green^®^ *Premix Ex Taq*™ (Tli RNaseH Plus) Kit (TaKaRa, Dalian, China) and the ABI Step One Plus^TM^ Real-Time PCR System (Foster City, CA, USA). The thermal cycling conditions were as follows: 95 °C for 30 s, followed by 40 cycles of 95 °C for 5 s, 60 °C for 30 s, and finally 95 °C for 15 s. The relative expression levels of 18 DEGs were calculated using the 2^−ΔΔCt^ method with three biological replicates. Statistical analysis was performed using IBM SPSS Statistics software (v26), and the significance of the difference between the mean values was tested using Duncan’s multiple range test (*p* < 0.05). Figures were generated using GraphPad Prism software (v8.0.2).

## 5. Conclusions

The first full-length transcriptome of half-highbush blueberry (*Vaccinium corymbosum/angustifolium* cultivar Northland) has been reported. Overall, 147,569 consensus transcripts with an average length of 2738 bp and an N50 of 3176 bp were obtained through SMRT sequencing. The SMRT sequencing data integrated with NGS data and the results of untargeted metabolomics analyses allowed us to accurately identify 270 functional genes (188 structural genes and 40 transporter genes) and 78 metabolites involved in the biosynthesis of flavonoids in mature fruits. A total of 4 delphinins are the core DEMs in fruits, while 14 functional genes (4 *Vc4CL*s, 3 *VcBZ1*s, 1 *VcUGT75C*1, 1 *VcAT*, 1 *VcUGAT*, 1 *VcGSTF*, and 3 *VcMATE*s) and 10 TFs (1 *VcMYB*, 2 *VcbHLH*s, 4 *VcWD40*s, and 3 *VcNAC*s) may play an important role in the biosynthesis of anthocyanins in blueberry fruits.

## Figures and Tables

**Table 1:** An overview of the blueberry full-length transcriptome.

PacBio Sequencing	
Subreads base (G)	9.4
Average subreads length (bp)	1496
FLNC	
CCS number	390,855
FLNC number	267,687
Mean length (bp)	2518
After correction by Illumina data	
Consensus number	147,569
Mean length/N50 (bp)	2738/3176
Mapped to the genome	138,908 (94.13%)
After correction and de-redundancy	
High-quality isoform number	63,425
Isoforms of known genes number	7584
Novel isoform of known genes number	49,005
Isoforms of novel genes number	6836
Novel genes number	5530

## Data Availability

The RNA-Seq and full-length transcriptome datasets from *Vaccinium* are deposited in the NCBI Sequence Read Archive (SRA) database (Bioproject ID: PRJNA1085853 and PRJNA1086160).

## Supplementary Materials

The following supporting information can be downloaded at: https://www.mdpi.com/article/10.3390/ijms25084137/s1.

## Abbreviations

FL: full length; NGS, next-generation sequencing; TF, transcription factor gene; EBG, early biosynthesis gene; LBG, late biosynthesis gene; PAL, phenylalanine ammonia lyase; C4H, cinnamate 4-hydroxylase; 4CL, 4-coumarate-CoA ligase; CHS, chalcone synthase; CHI, chalcone isomerase; F3H, flavanone-3-hydroxylase; FLS, flavonol synthase; DFR, dihydroflavonol 4-reductase; ANS, anthocyanidin synthase; LAR, leucoanthocyanidin reductase; OMT, O-methyltransferase; AT, acyltransferase; UFGT, UDP Glc-flavonoid 3-O-glucosyltransferase; MBW, MYB-bHLH-WD40; PCA, principal component analysis; OPLS-DA, orthogonal partial least-squares discrimination analysis; DEM, differentially expressed metabolite; DEG, differentially expressed gene; AS, alternative splicing; C3′H, 5-O-4-coumaroyl-D-quinate 3′-monooxygenase; COMT, caffeic acid 3-O-methyltransferase/acetylserotonin O-methyltransferase; F5H, ferulate-5-hydroxylase; CCR, cinnamoyl-CoA reductase; CAD, cinnamyl-alcohol dehydrogenase; HCT, shikimate O-hydroxycinnamoyltransferase; CSE, caffeoylshikimate esterase; CCoAOMT, caffeoyl-CoA O-methyltransferase; F3′H, flavonoid 3′-monooxygenase; F3′5′H, flavonoid 3′,5′-hydroxylase; FG2, flavonol 3-O-glucoside L-rhamnosyltransferase; F3GalT, flavonol 3-O-β-D-galactosyltransferase; UGT73C6, flavonol 3-O-L-rhamnoside-7-O-glucosyltransferase; LDOX, leucoanthocyanidin dioxygenase; HIDH, 2-hydroxyisoflavanone dehydratase; PGT1, phlorizin synthase; C12RT1, flavanone 7-O-glucoside 2″-O-beta-L-rhamnosyltransferase; CYP81E, isoflavone/4′-methoxyisoflavone 2′-hydroxylase; PTS, pterocarpan synthase; 3GGT, anthocyanidin 3-O-glucoside 2″-O-glucosyltransferase; BZ1, anthocyanidin 3-O-glucosyltransferase; GT1, anthocyanidin 5,3-O-glucosyltransferase; UGAT, cyanidin 3-O-glucoside 2″-O-glucuronosyltransferase; UGT75C1, anthocyanidin 3-O-glucoside 5-O-glucosyltransferase; GST, glutathione-S-transferase; MATE, multidrug and toxic compound extrusion transporter; ABC, ATP-binding cassette transporter.
