# Supplementary material for: Integrated Untargeted Metabolome, Full-Length Sequencing and Transcriptome Analyses Reveal the Mechanism of Flavonoid Biosynthesis in Blueberry (Vaccinium spp.) Fruit
Source: Int J Mol Sci. 2024 Apr 9;25(8):4137. doi: 10.3390/ijms25084137 (PMC11050320; doi:10.3390/ijms25084137)
Supplement: Supplementary file 1 [file ijms-25-04137-s001.zip › Figure S1. Heatmap showing relative abundance of flavonoids and phenylpropanoids in Ex, Se and Sa samples.pdf]

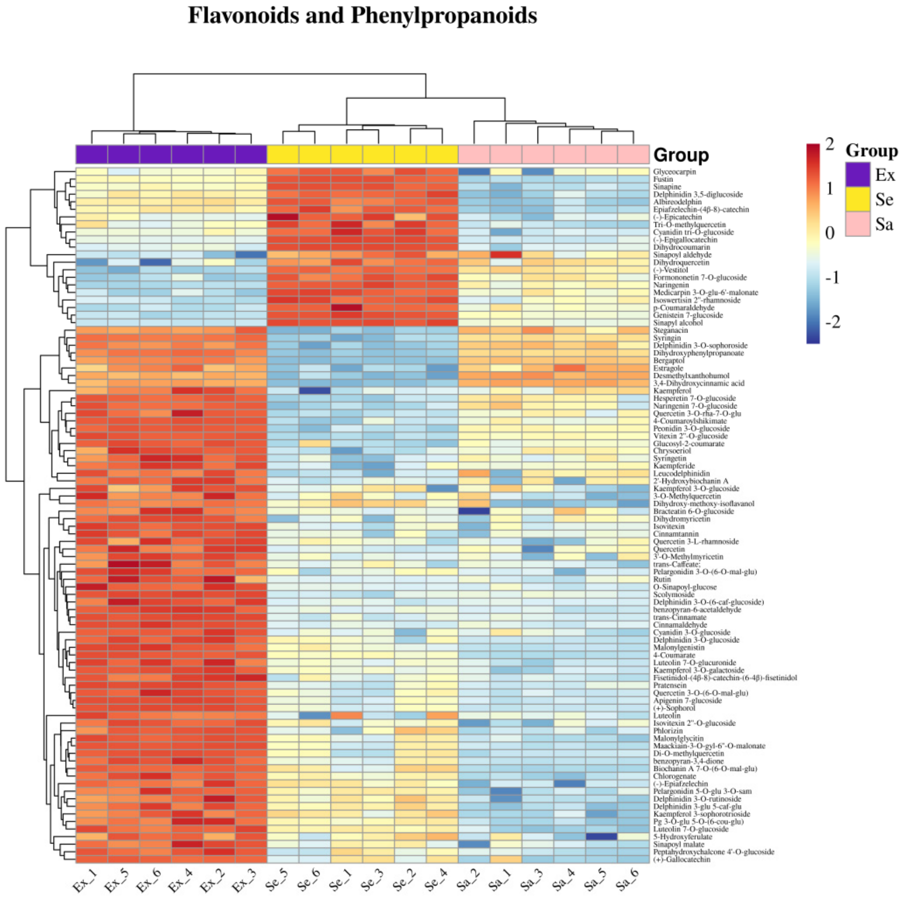

Figure S1. Heatmap showing relative abundance of flavonoids and phenylpropanoids in Ex, Se and Sa samples. Red and blue indicate up-regulation and down-regulation, respectively.
