# Supplementary material for: Integrated Untargeted Metabolome, Full-Length Sequencing and Transcriptome Analyses Reveal the Mechanism of Flavonoid Biosynthesis in Blueberry (Vaccinium spp.) Fruit
Source: Int J Mol Sci. 2024 Apr 9;25(8):4137. doi: 10.3390/ijms25084137 (PMC11050320; doi:10.3390/ijms25084137)
Supplement: Supplementary file 1 [file ijms-25-04137-s001.zip › Figure S3. Gene structural information of full-length transcriptome..pdf]

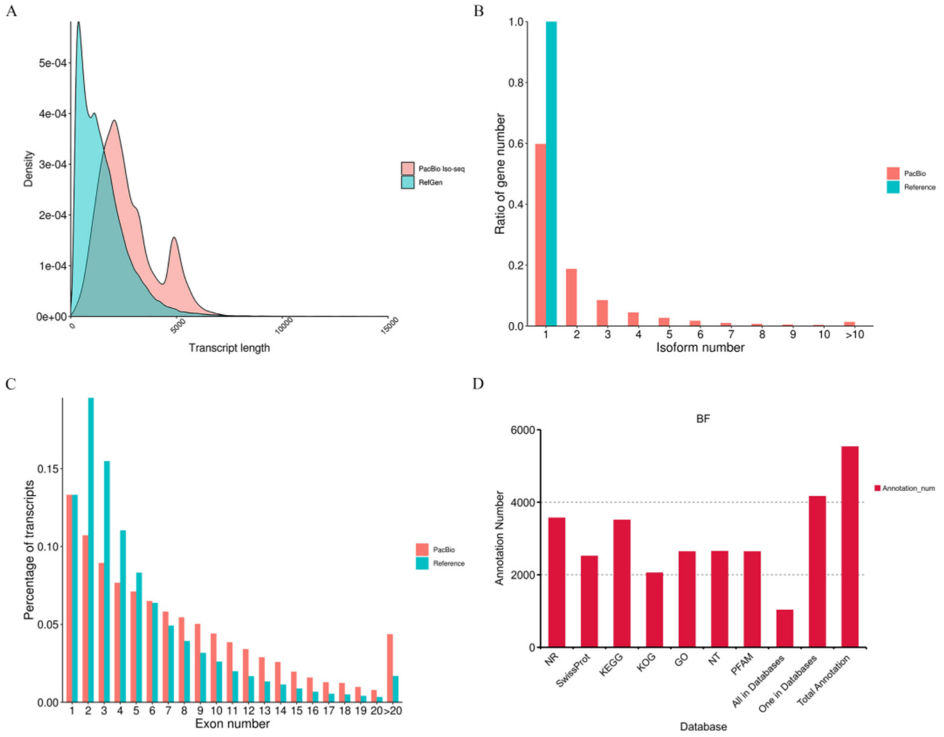

Figure S3. Gene structural information of full-length transcriptome. A. Transcript length density distribution plot. B. Distribution map of the number of transcripts corresponding to each gene. C. Distribution diagram of the number of exons corresponding to each transcript. D. Statistical chart of annotation results of seven major databases.
