# Supplementary figures and images for: Integrated Untargeted Metabolome, Full-Length Sequencing and Transcriptome Analyses Reveal the Mechanism of Flavonoid Biosynthesis in Blueberry (Vaccinium spp.) Fruit
Source: Int J Mol Sci. 2024 Apr 9;25(8):4137. doi: 10.3390/ijms25084137 (PMC11050320; doi:10.3390/ijms25084137)

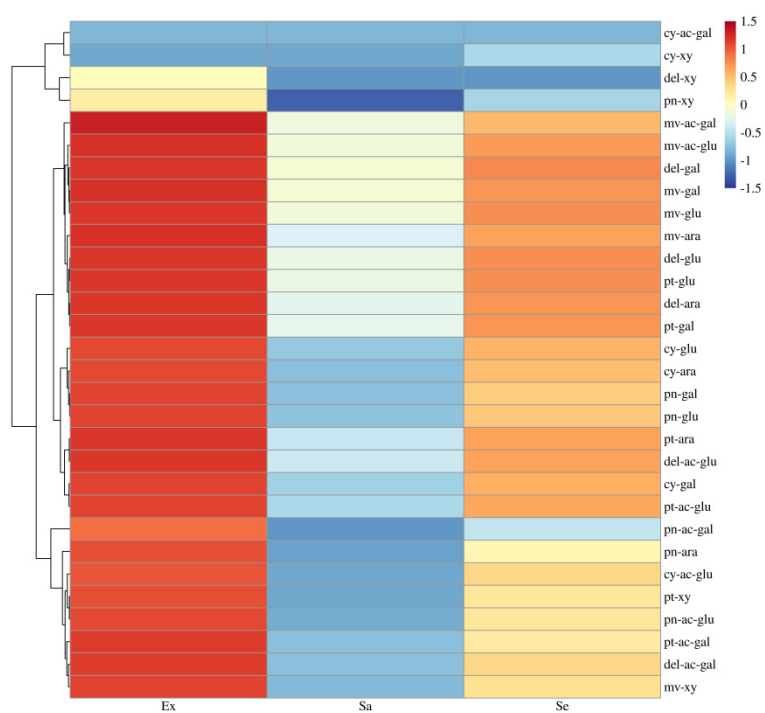

Figure S2. Heatmap of the contents of 30 anthocyanins in blueberry fruit tissues.

Supplement: Supplementary file 1 [file ijms-25-04137-s001.zip › Figure S2. Heatmap of the contents of 30 anthocyanins in blueberry fruit tissues.pdf]
